# Direct Interleukin-6 Inhibition Blunts Arterial Thrombosis by Reducing Collagen-Mediated Platelet Activation

**DOI:** 10.1161/ATVBAHA.125.322533

**Published:** 2025-06-19

**Authors:** Stefano Ministrini, Luca Liberale, Yustina M. Puspitasari, Jiaying Han, Kilian Kirmes, Leonhard Paul Unkelbach, Amedeo Tirandi, Rebecca Niederberger, Susan Bengs, Jürg H. Beer, Fabrizio Montecucco, Peter Libby, Thomas F. Lüscher, Dario Bongiovanni, Giovanni G. Camici

**Affiliations:** Center for Molecular Cardiology, University of Zurich, Schlieren, Switzerland (S.M., Y.M.P., A.T., R.N., S.B., J.H.B., T.F.L., G.G.C.).; First Clinic of Internal Medicine, Department of Internal Medicine, University of Genoa, Italy (L.L., A.T., F.M.).; IRCCS Ospedale Policlinico San Martino Genoa-Italian Cardiovascular Network (L.L., F.M.).; Department of Internal Medicine I, School of Medicine, University Hospital Rechts der Isar, Technical University of Munich, Germany (J.H., K.K.).; Department of Internal Medicine I, Cardiology, University Hospital Augsburg, University of Augsburg, Germany (L.P.U., D.B.).; Division of Cardiovascular Medicine, Brigham and Women’s Hospital, Harvard Medical School, Boston, MA (P.L.).; Royal Brompton & Harefield Hospitals and Cardiovascular Academic Group, King’s College, London, United Kingdom (T.F.L.).; Department of Research and Education, University Hospital Zurich, Switzerland (G.G.C.).

**Keywords:** inflammation, interleukin-6, risk, thrombosis, ziltivekimab

## Abstract

**BACKGROUND:**

Recent clinical trials demonstrated a reduction in biomarkers of thrombosis and inflammation in patients with very high cardiovascular risk treated with the anti–IL-6 (interleukin 6) monoclonal antibody ziltivekimab. However, if and how direct IL-6 inhibition exerts antithrombotic effects remains unknown. This translational project aimed to investigate the effect of direct IL-6 inhibition on experimental arterial thrombus formation and its underlying cellular mechanisms.

**METHODS:**

Three-month-old C57BL/6J male and female mice received very low dose lipopolysaccharide for 4 weeks; in addition to lipopolysaccharide, during the fourth week, mice were randomized to receive either anti-mouse IL-6 monoclonal antibody 200 μg or IgG1 isotype control. Thrombosis of the right common carotid artery was induced by endothelial-targeted laser injury. Coagulation factors and platelet reactivity were assessed in treated mice and controls. Platelets were isolated from whole blood and their reactivity to different chemical stimuli was measured by fluorescence-activated cell sorting. Additionally, whole blood samples from patients with a history of percutaneous coronary intervention were incubated ex vivo with either ziltivekimab biosimilar or IgG1 isotype control. Platelet reactivity at rest and in response to diverse chemical stimuli was quantified by fluorescence-activated cell sorting.

**RESULTS:**

Mice with low-grade chronic inflammation treated with anti–IL-6 monoclonal antibody displayed significantly blunted thrombus formation, without any significant difference in coagulation factors. Ex vivo stimulation with Collagen-rP (collagen-related peptide) significantly activated platelets isolated from control mice but not those obtained from mice treated with anti–IL-6 monoclonal antibody. Similarly, platelet reactivity from patients with previous percutaneous coronary intervention fell significantly after ex vivo treatment with ziltivekimab biosimilar.

**CONCLUSIONS:**

Direct IL-6 inhibition blunts thrombus formation by reducing collagen-induced platelet activation. These findings offer a potential mechanistic explanation for the results observed in the RESCUE trial and support the rationale of the ongoing ZEUS trial (Ziltivekimab Cardiovascular Outcome Study).

**Highlights**

- Atherothrombosis and acute inflammation are key processes in cardiovascular disease and major acute cardiovascular events.
- The role of IL-6 (interleukin 6) in the pathogenesis of cardiovascular disease is well-established. However, no pharmacological treatment targeting this cytokine has been demonstrated effective in preventing major acute cardiovascular events at the present time.
- Our results show that direct inhibition of IL-6 can prevent arterial thrombosis in a murine model of chronic low-grade inflammation, by blunting platelet aggregation in response to subintimal collagen exposure. These results are confirmed ex vivo in patients with very high cardiovascular risk.
- These results support the potential clinical application of direct IL-6 inhibition for the prevention of major acute cardiovascular events in subjects with very high risk for cardiovascular disease, as already under investigation in advanced clinical trials.

Acute cardiovascular and cerebrovascular diseases, such as acute myocardial infarction or acute ischemic stroke, represent common causes of death and disability worldwide.^1,2^ Multiple, sometimes overlapping, mechanisms underlie these conditions, including impaired vascular reactivity to hemodynamic stress, arterial stenosis due to atherosclerosis, or arterial wall hyperplasia compromised microcirculatory function and eventually superimposed arterial thrombosis leading to vascular occlusion.^3^ Arterial thrombosis arises from the concurrent presence of endothelial injury, platelet activation, and hypercoagulability.^4^ Systemic inflammation promotes the occurrence of these conditions, serving as a well-established risk factor for both cardiovascular disease and cerebrovascular disease. Consequently, inflammatory mediators represent promising targets for preventing acute cardiovascular disease and cerebrovascular disease.^5^

IL-6 (interleukin 6) is a cytokine with pleiotropic effects on host immune response to pathogens, inflammation, and hematopoiesis. It is produced and released by many cell types, including T lymphocytes, in response to infections and tissue injury, contributing to host defense and inflammation.^6^ IL-6 levels predict vascular events in primary and secondary prevention with accuracy comparable to that of hsCRP (high-sensitivity C-reactive protein).^7^ Furthermore, Mendelian randomization studies suggest that genetic variants in the IL-6 receptor signaling pathway are associated with the lifetime risk of coronary heart disease.^8^

From the clinical perspective, a single administration of a blocking monoclonal antibody (mAb) against the IL-6 receptor (tocilizumab) before coronary intervention in patients with non–ST-segment–elevation myocardial infarction leads to reduced circulating levels of hsCRP and troponin T.^9^ More recently, the phase 2 RESCUE trial confirmed a reduction in biomarkers of inflammation and thrombosis in patients with chronic kidney disease and elevated levels of hsCRP treated with the IL-6-blocking mAb ziltivekimab.^10^ The efficacy of ziltivekimab on cardiovascular events in such patients is currently being investigated in ZEUS (Ziltivekimab Cardiovascular Outcome Study) a large, long-term phase 2 trial.^11^ Based on the evidence discussed above, this translational project aimed to investigate whether direct IL-6 inhibition affects arterial thrombus formation and the underlying cellular mechanisms to provide a deeper insight into the mechanistic background of ongoing clinical trials.

## Methods

The data that support the findings of this study, as well as analytic methods and study materials, are available from the corresponding author upon reasonable request.

### Animals

Three-month-old C57BL/6J wild-type male and female mice received very low dose lipopolysaccharide intraperitoneally for 4 weeks to induce a state of chronic low-grade inflammation, as previously described.^12,13^ During the past week, mice were randomized to receive in addition to lipopolysaccharide either anti-mouse IL-6 mAb (BE0046, Bio X Cell, PA) 200 μg IP every other day or the same amount of IgG1 isotype control (BE0290, Bio X Cell, PA), as previously described.^14,15^ Ten-week old animals were purchased from Charles River Laboratories (Lyon, France) and acclimatized to the animal facility located in the Schlieren Campus of the University of Zurich for 2 weeks. Animals were raised in optimal hygiene conditions, with a stable temperature of 20 to 22 °C, and fed with an ad libitum chow diet (Kliba Nafag 3336, 17 MJ/kg; Kliba Nafag, Kaiseraugst, Switzerland). Mice were sequentially numbered and allocated to the intervention groups using a casual number generator (https://www.calculator.net/random-number-generator.html). Only 1 experimenter (S.B.) knew the allocation of the animals. The allocation template was disclosed after statistical analysis. Animal experiments were approved by the ethics committee of the Canton Zurich Veterinary Office (license ZH148/2023). Animals meeting humane end points were euthanized, and collected data were excluded from the analysis. Sex differences and their interaction with the treatment were not analyzed in the current study.

### Photochemically Induced Arterial Thrombosis

Both groups of mice underwent endothelial-targeted photochemical injury of the right common carotid artery to induce arterial thrombosis. The detailed description of this technique has been previously reported.^16^ Briefly, Rose Bengal (62.6 mg/kg of body weight) was administered by tail-vein injection in anesthetized mice. Mice were placed in a supine position under a dissecting microscope, and the right common carotid artery was exposed by a midline cervical incision. A Doppler-flow probe was applied around the right common carotid artery and connected to a flowmeter. Five to 10 minutes after Rose Bengal injection, a 1.5 mW green light laser was directed to the site of injury at 6 cm from the artery, until thrombosis occurred. Occlusion was defined as blood flow below 0.1 mL/min for at least 1 minute, and the time-to-occlusion was used as a measure of arterial thrombosis efficacy. Cyclic flow variations were recorded, and thrombus embolization was defined as an increase of blood flow to above 0.1 mL/min after a previous decrease below said level lasting <1 minute. Clot embolization episodes reflect thrombus stability.^16,17^

### Platelet Reactivity Assay in Murine Samples

Blood samples were terminally drawn by cardiac puncture under anesthesia and collected in sodium citrate 0.1 M. A 50 μL aliquot of whole blood was employed for cell count (ScilVet ABCplus, Horiba, Kyoto, Japan). Platelet-rich plasma was obtained by centrifugation at 200*g* for 8 minutes, then incubated for 10 minutes at 37 °C with the addition of 0.02 U/mL of potato apyrase (A6535; Sigma-Aldrich, MO) and 1 μL/mL prostaglandin E1 (538903; Merck, Darmstadt, Germany) as described before.^17^ Platelets were isolated from platelet-rich plasma by centrifugation (800*g* for 15 minutes), then washed twice in modified Tyrode-HEPES buffer (5 mmol/L HEPES, 137 mmol/L NaCl, 0.42 mmol/L NaH_2_PO_4_, 2 mmol/L KCl, 12 mmol/L NaHCO_3_, 5.5 mmol/L glucose, 0.35% bovine serum albumin, and pH 7.35) with 1 μg/mL prostaglandin E1. Afterward, washed platelets were resuspended in modified Tyrode-HEPES buffer with 1 mmol/L CaCl_2_ and 1 mmol/L MgCl2 and activated with ADP (10 μM, 01905; Sigma-Aldrich, St. Louis, MO), Collagen-rP (collagen-related peptide; 20 μg/mL, CRP-XL; Camb Collabs, Littleport, United Kingdom) or thrombin from human plasma (0.1 U/mL; T6884; Merck, Darmstadt, Germany). Agonists’ concentrations were specified according to previous ex vivo platelets aggregation assay.^12,16,18^ In parallel, washed platelets were labeled with fluorescent anti-mouse antibodies against CD (cluster derived) 41 (1.3 μg/mL; BioLegend, San Diego, CA), CD62P (0.5 μg/mL; eBioscience, San Diego, CA) and activated Gp (glycoprotein) IIb/IIIa (JON/A, 0.75 μg/mL; EMFRET Analytics, Würzburg, Germany). CD41 was employed as platelet-specific membrane marker, whereas CD62P and activated GpIIb/IIIa were employed as activation markers, as previously described.^19–21^ Platelets reactivity will be measured by fluorescence-activated cell sorting (FACS–LSR II Fortessa 4 L; BD & Company, Franklin Lakes, NJ), acquiring 10 000 CD41-positive events, as mean fluorescence intensity of CD62P and activated GpIIb/IIIa.

### Patients

Fifteen (15) patients subjected to percutaneous coronary intervention 6 months earlier were enrolled in this study. According to the guidelines of the European Society of Cardiology, all these subjects qualify as very high risk of major adverse cardiovascular events.^22^ The study was approved by the ethics committee of Klinikum rechts der Isar, Munich, Germany (no. 2023-77-S-SR) and was conducted in accordance with the 1964 Declaration of Helsinki and its later amendments. All patients signed a written informed consent. Venous blood from each patient was collected in the morning after at least 8 hours of fasting and stored in tubes with 3.2% citrate. Whole blood samples were preincubated with 1.5 µg/mL ziltivekimab biosimilar (PX-TA-1613-100, Proteogenix, Schiltigheim, France)^23^ or IgG1 isotype (PTX-17897, Proteogenix, Schiltigheim, France) for 30 minutes at room temperature.

### Platelets Reactivity Assay in Human Samples

Whole blood samples were incubated with ADP (5 μM, 01905; Sigma-Aldrich, St. Louis, MO), Collagen-rP (2 μg/mL, CrP-XL; Camb Collabs, Littleport, United Kingdom), and TRAP-6 (thrombin receptor agonist peptide-6) amide (10 μM, 4031274; Bachem, Bubendorf, Switzerland) at room temperature. In parallel, samples were stained with anti-human antibodies against CD41 (1 μg/mL, 303730; Bio Legend, San Diego, CA) and CD62P (7.5 μg/mL, 550561; BD Biosciences; Franklin Lakes, NJ). After incubation in the dark for 30 minutes, the whole blood samples were fixed, and erythrocytes were lysed with Lyse/Fix Buffer (558049; BD Biosciences, Franklin Lakes, NJ) for 10 minutes at room temperature. CD41 was employed as a platelet-specific membrane marker, whereas CD62P was employed as activation markers, as previously described.^19,20^ Platelet reactivity was measured by fluorescence-activated cell sorting (Gallios, Beckman Coulter, Brea, CA), acquiring 10 000 CD41-positive events, as mean fluorescence intensity of CD62P.

### Statistical Analysis

Statistical analyses were performed using GraphPad Prism 6 software (GraphPad Software Inc, La Jolla, CA). The ROUT method was employed to identify outliers for a set Q=1%. Outliers were a priori excluded from inferential analyses (n=2 mice, both in the isotype control group). Normality of data was assessed by the Kolmogorov-Smirnov test. For the comparison of means, we used the paired and unpaired 2-tailed Student *t* test for normally distributed variables, or the Mann-Whitney *U* test and the paired Wilcoxon test for non-normally distributed variables. To compare ≥3 groups, 1-way and 2-way ANOVA with Bonferroni post hoc test for multiple comparisons were employed as appropriate. A *P*<0.05 was considered statistically significant.

Methods description for hemostasis assessment in mice and in vitro cell experiments (Figure S1) are reported in the Supplementary Methods. For a summary of methods, please refer to the Major Resources Table in the Supplemental Material.

## Results

**Figure 1.**
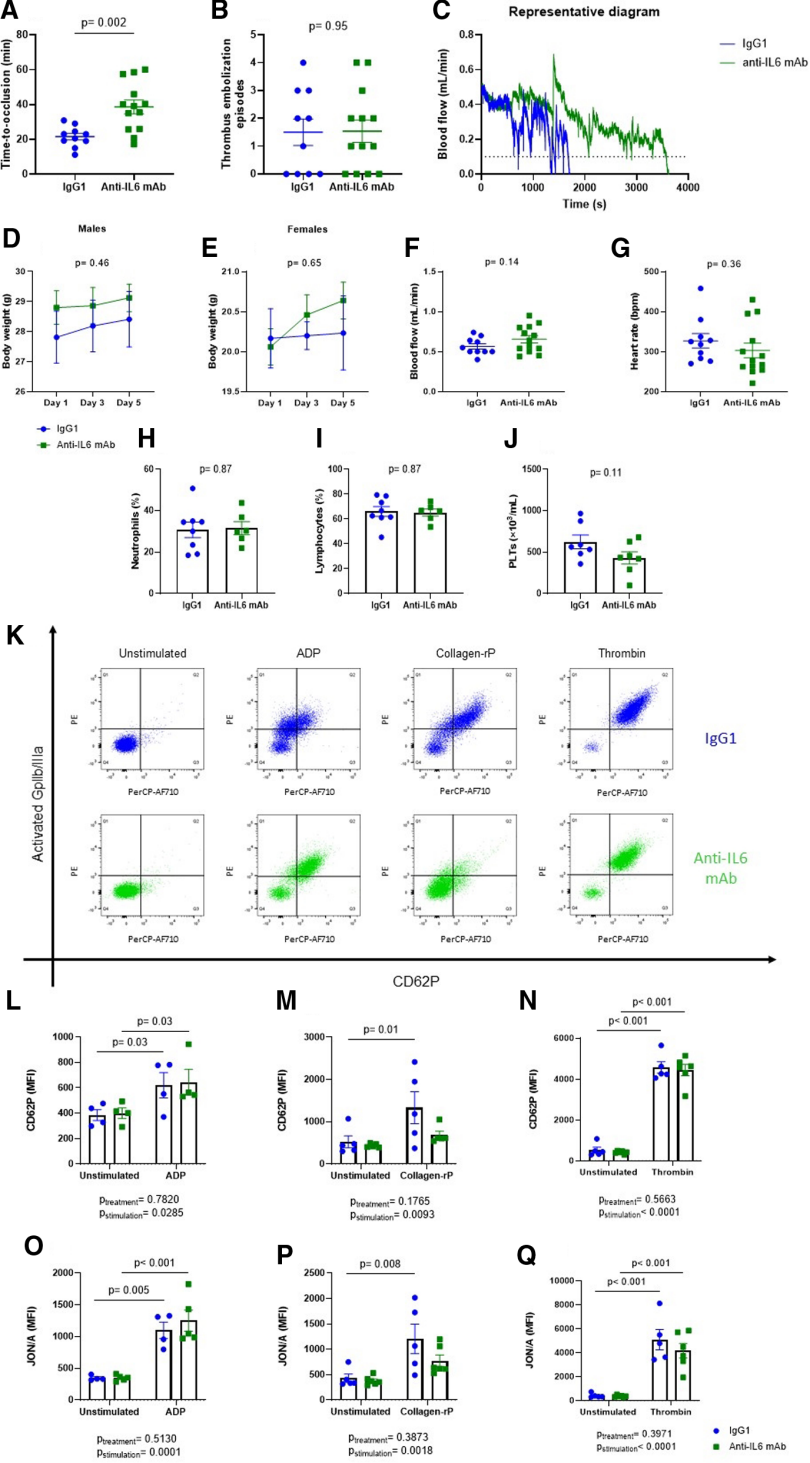
**Effects of direct IL-6 (interleukin 6) inhibition on carotid photochemical thrombosis and platelet reactivity in mice.** Chronic low-grade inflammation was induced by intraperitoneal administration of lipopolysaccharide; then mice were treated with either anti–IL-6 monoclonal antibody (mAb) or IgG1 isotype control. Comparison of time-to-occlusion of the right common carotid artery (**A**) and embolization episodes (**B**) between treated and control mice. Representative trace of mean blood flow until occlusion in the 2 study groups (**C**). Comparison of baseline physiological parameters between treated and untreated mice: body weight (**D** and **E**), blood flow in the right common carotid artery (**F**), heart rate (**G**), neutrophils (**H**), lymphocytes (**I**), and platelets count (**J**). Representative picture of flow-cytometry analysis of platelets activation upon stimulation with different activators (ADP, Collagen-rP [collagen-related peptide], and thrombin) in treated and control mice (**K**). Expression of CD62P (P-selectin) after stimulation with different activators (ADP, Collagen-rP, and TRAP [thrombin receptor agonist peptide]) in treated and control mice (**L** through **N**). Expression of activated Gp (glycoprotein) IIb/IIIa (JON/A) after stimulation with different activators (ADP, Collagen-rP, and TRAP) in treated and control mice (**O** through **Q**). Data are presented as mean±SEM. Student *t* test (**A**, **F** through **J**), Mann-Whitney *U* test (**B**), and 2-way repeated measures ANOVA (**L** through **Q**); n=10 mice (**A** through **G**), n=6 mice (**H** through **J**) and n=5 mice (**L** through **Q**). CD indicates cluster derived; MFI, mean fluorescence intensity; and PerCP, peridinin-chlorophyll-protein.

**Figure 2.**
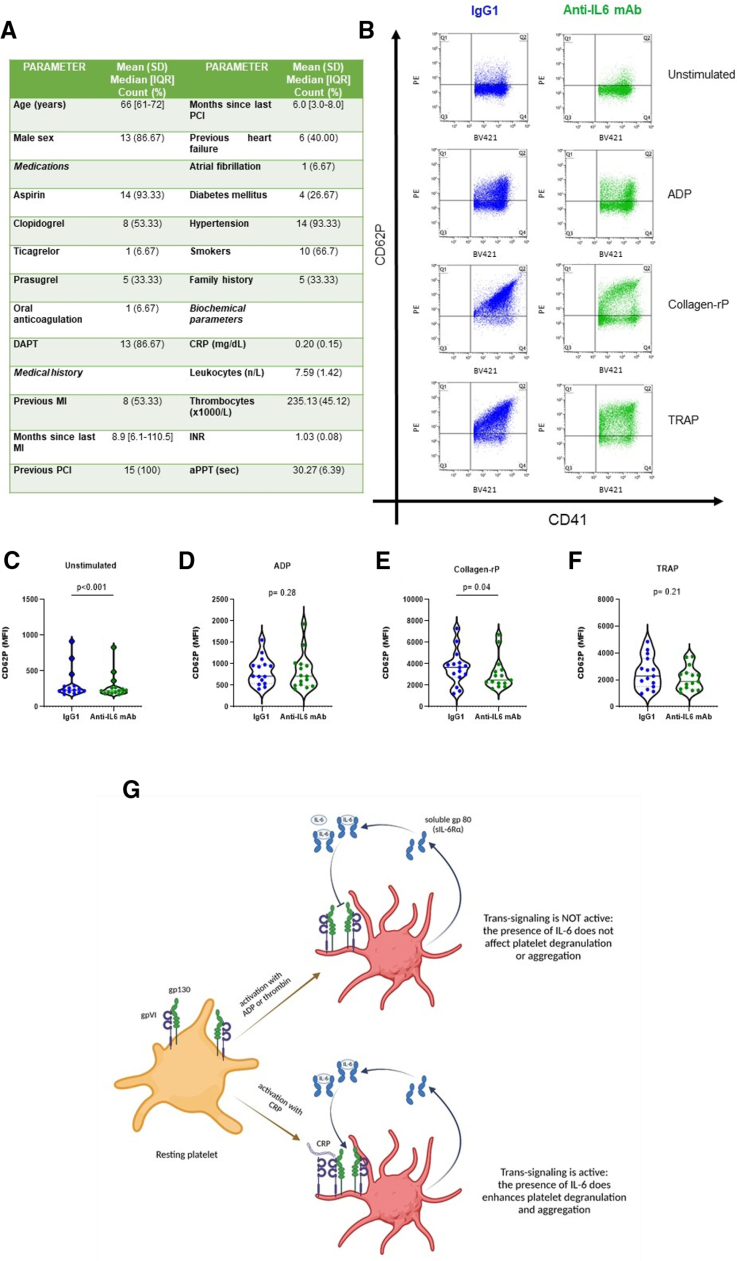
**Effects of direct IL-6 (interleukin 6) inhibition on platelet reactivity of patients with very high cardiovascular risk.** Platelets of very high cardiovascular risk patients were treated ex vivo with either ziltivekimab biosimilar or IgG1 isotype control. Demographic, clinical, and biochemical characteristics of donor subjects (**A**). Representative picture of flow-cytometry analysis of treated and control platelets after stimulation with different activators (ADP [adenosine diphosphate], Collagen-rP [collagen-related peptide], and TRAP [thrombin receptor agonist peptide]; **B**). Comparison of CD62P expression in treated and control platelets after stimulation with different activators (ADP, Collagen-rP, and TRAP; **C** through **F**). Graphical summary (**G**). Data are presented as median with 25–75 percentile. Paired Wilcoxon test (**C** and **D**) and paired Student *t* test (**E** and **F**); n=15 patients. aPTT indicates activated partial thromboplastin time; CD, cluster derived; CRP, C-reactive protein; DAPT, dual antiplatelet therapy; Gp, glycoprotein; INR, international normalized ratio; IQR, interquartile range; mAb, monoclonal antibody; MFI, mean fluorescence intensity; MI, myocardial infarction; PCI, percutaneous coronary intervention; and PE, phycoerythrin.

Mice treated with anti–IL-6 mAb displayed a significantly longer time-to-occlusion, as shown in Figure 1A. Thrombus embolization episodes did not differ between the 2 groups (Figure 1B). A representative diagram of blood flow in the right common carotid artery during the experiment in both anti–IL-6 mAb treated animals and controls is reported in Figure 1C. Physiological variables, such as body weight, blood flow in the carotid artery at baseline, heart rate at baseline, and white blood cell count did not differ significantly (Figure 1D through 1I). Unexpectedly, coagulation parameters (ie, prothrombin time, thrombin/antithrombin III complex, plasma and vascular tissue factor, plasma and vascular plasminogen activator inhibitor 1) did not differ between the 2 groups (Figure S1A through S1H). Furthermore, in vitro experiments in human aortic endothelial cells and THP-1 cells confirmed that the expression of endothelial- and monocyte-derived prothrombotic factors is not affected by the treatment with anti–IL-6 mAb (Figure S1I through S1P). Thus the potential involvement of platelets was investigated by assessing platelet reactivity upon stimulation with different activators (Figure 1J). After stimulation with Collagen-rP, platelets were significantly activated in isotype control-treated mice, but not in mice treated with anti–IL-6 mAb (Figures 1L and 1O). Stimulation with ADP (Figures 1K and 1N) and TRAP (Figures 1M and 1P) induced a significant activation in both IL-6 mAb group and the isotype control group. To increase the translational relevance of the results observed in murine platelets, we assessed the reactivity of platelets isolated from patients with very high cardiovascular risk and ex vivo treated with ziltivekimab biosimilar. Characteristics of enrolled patients are summarized in Figure 2A. Patients had a median age of 66 years and were predominantly men with systemic arterial hypertension. Of note, all patients had ongoing antithrombotic therapy and 87% received dual antiplatelet therapy. Consistently with what was observed in mice, platelets treated with ziltivekimab biosimilar displayed a significantly lower expression of CD62P after stimulation with Collagen-rP (Figure 2B, 2C, and 2E), without significant differences upon stimulation with either ADP or TRAP (Figure 2B, 2D, and 2F).

## Discussion

As demonstrated in several clinical trials, targeting inflammation can reduce very high residual cardiovascular risk in patients with acute coronary syndromes or stroke.^5^ More recently, the RESCUE trial reported reduced biomarkers of inflammation and thrombosis in high cardiovascular risk patients treated with the anti–IL-6 mAb ziltivekimab. Based on these observations, a large-scale cardiovascular outcomes trial (ZEUS) is underway. However, if and how direct IL-6 ligand inhibition may provide antithrombotic effects remains unknown. This project investigated the effect of direct IL-6 inhibition on arterial thrombus formation and the underlying cellular mechanisms. Using a murine model of arterial thrombosis, we found blunted thrombus formation in mice receiving direct IL-6 blockade. Of note, before treatment with anti–IL-6 mAb, a state of chronic low-grade inflammation was induced in mice administering low-dose lipopolysaccharide, to mimic the baseline conditions of patients included in the RESCUE trial, who presented chronic kidney disease and systemic inflammation as assessed by hsCRP. IL-6 is secreted by several different cells, such as monocytes, fibroblasts, and endothelial cells, typically after IL-1 stimulation.^24^ Once secreted in the bloodstream, IL-6 has a range of pleiotropic effects on several different cells, tissues, and organs. Among them, IL-6 induces the liver to synthesize a group of proteins, that is, acute phase proteins, which include CRP, serum amyloid A, and some coagulation cascade components.^25^ Furthermore IL-6 has a direct effect on endothelial cells, promoting the production of several cytokines and chemokines and eventually initiating the coagulation cascade.^26^ However, investigation of coagulation factors in the murine model (data not shown) retrieved neutral results, suggesting that the observed antithrombotic effects of direct IL-6 blockade are not related to decreased production coagulation factors or fibrinolysis.

Platelets play a pivotal role in arterial thrombus formation upon endothelial damage; therefore, we assessed their reactivity as a possible determinant of the observed antithrombotic effect. Interestingly, we found that platelet reactivity was selectively reduced in response to Collagen-rP, but not ADP or TRAP, in mice treated with IL-6 antibody. This mechanism is particularly relevant to the murine model of arterial thrombosis employed herein, as well as to acute atherothrombotic events. Indeed, this model is based on exposure of collagen in the basement membrane in the subendothelial layer, similar to what occurs after atherosclerotic plaque erosion.^27^ To increase the translational relevance of our results, we incubated platelets from very high cardiovascular risk patients ex vivo with ziltivekimab biosimilar or IgG1 isotype control. Similar to what was observed in mice, ziltivekimab selectively inhibited platelet reactivity in response to Collagen-rP, but not ADP or TRAP.

The interaction between the vascular wall and platelets is crucial in arterial thrombus formation, but the role of IL-6 in this process is still unclear. Endothelial cells release IL-6 under several stressful conditions, including exposure to lipopolysaccharide as in the present study.^28,29^ On the other hand, platelets induce the release of IL-6 from endothelial cells through the secretion of soluble CD 40 ligand.^30^ IL-6 may, therefore, play a pivotal role in the amplification and propagation of the thrombosis cascade. IL-6 signaling is a complex process that can occur in 3 possible modalities: classic signaling, trans-signaling, and transpresentation.^31^ With classic signaling, the cytokine binds its transmembrane receptor gp80 and the IL-6/gp80 complex binds the transmembrane adaptor gp130, activating a signal transduction cascade, mainly through the STAT3 (signal transducer and activator of transcription 3).^32^ In the trans-signaling, IL-6 binds its soluble receptor (sIL-6R [soluble IL-6 receptor]), constituted by a 55 kDa fraction of gp80, and the IL-6/sIL-6R complex eventually binds the adaptor gp130. Finally, in the transpresentation, IL-6 binds gp80 on the surface of a cell and the IL-6/gp80 complex binds gp130 on the surface of a different cell, mediating a cell-to-cell interaction.^24^ Resting platelets do not express gp80 on their membrane, but they express gp130 and are thus theoretically responsive to trans-signaling. Experimental evidence shows that, upon stimulation with thrombin, platelets secrete sIL-6R and, in the presence of IL-6, trans-signaling activation induces the expression of gp80 through granules mobilization, thus generating an autocrine activation loop (Figure 2G).^33–35^ Although trans-signaling has no effect on platelet degranulation or aggregation by itself,^35^ our results suggest that this autocrine activation loop could have a functional role upon stimulation with collagen. This effect was previously hypothesized by Houck et al^36^ and Senchenkova et al,^37^ who described the physical proximity and functional cooperation of gp130 and the collagen receptor gpVI on platelet membrane. The results presented herein provide a functional demonstration of a cross-talk between collagen and IL-6 pathways in platelets. Furthermore, they propose a potential mechanism to explain the expected reduction in cardiovascular risk with direct IL-6 inhibition and support the investigation of direct IL-6 inhibition in clinical practice. In this regard, direct IL-6 blockade may have pleiotropic effects on cardiovascular risk, over and beyond platelet aggregation, by affecting atherosclerosis progression and megakaryocyte maturation, among others.^38,39^ Interestingly, Marino et al^35^ observed that the P2Y12 receptor inhibitor cangrelor inhibits the release of sIL-6R from platelets, suggesting that direct IL-6 inhibition could have an overlapping function with P2Y12 receptor inhibitors, a class of drugs commonly employed in dual antiplatelet therapy. In a future perspective, direct IL-6 inhibition could help refine antiplatelet therapy in patients with concomitant high bleeding and high thrombotic risk. In this regard, a vast majority of patients with very high cardiovascular risk receive antiplatelet drugs to prevent the onset or recurrence of major atherothrombotic events. Consistently, >90% of patients recruited for the ex vivo experiment were treated with antiplatelet drugs. The demonstration, although ex vivo, of an effect for direct IL-6 inhibition on top of antiplatelet therapy has great translational relevance, as it supports the potential use of ziltivekimab as an additional treatment for the prevention of major atherothrombotic events.

This study presents some limitations. First, data from very high cardiovascular risk patients were obtained by ex vivo incubation of blood samples with ziltivekimab and, therefore, our results do not account for the contribution of other organs and tissues, such as vascular wall and liver, whose function could be affected by the direct IL-6 inhibition in vivo. However, with this approach, we could highlight the direct effect of IL-6 inhibition on platelets excluding possible off-target effects. Although we excluded their contribution in the murine model, some differences may still exist between real patients and mice. As regards the murine model of arterial thrombosis, previous publications highlighted that mechanisms of photothrombosis could be partially independent of platelet activation.^40^ However, our group successfully investigated platelet dysfunction in a murine model of Hutchinson-Gilford progeria syndrome using this very same method.^21^ Furthermore, although whole blood was treated with ziltivekimab, only platelets were analyzed. However, whole blood contains several other cells whose function can be modified by direct IL-6 blockade and which can, in turn, interact with platelets. Finally, molecular mechanisms linking collagen and IL-6 signaling in platelets were not further investigated in this paper, a subject for future follow-up studies.

## Conclusions

Direct IL-6 inhibition attenuates arterial thrombosis in a murine model of endothelial damage and chronic low-grade inflammation. This effect can be attributed to a reduced platelets reactivity to collagen, as observed in both mice and patients with very high cardiovascular risk. These results support the possible use of direct IL-6 inhibition to reduce residual risk and may complement the ongoing clinical trials on this topic.

## Article Information

### Acknowledgments

G.G. Camici and L. Liberale conceived the project. S. Ministrini, L. Liberale, S. Bengs, and Y.M. Puspitasari performed in vivo experiments. D. Bongiovanni, J. Han, and K. Kirmes performed ex vivo experiments in human platelets. S. Ministrini and A. Tirandi drafted the paper and performed statistical analysis. S. Ministrini, Y.M. Puspitasari, S. Bengs, and R. Niederberger performed biomarkers measurements and in vitro experiments. D. Bongiovanni, S. Bengs, and F. Montecucco reviewed and finalized the paper. P. Libby, J.H. Beer, T.F. Lüscher, and G.G. Camici supervised the project and made critical changes to the manuscript. G.G. Camici provided funding for the project. All authors approved the final manuscript.

### Sources of Funding

This work was funded by a grant from the Swiss Heart Foundation (FF24041/2024) and the Swiss National Science Foundation (grant number 197510) to G.G. Camici.

### Disclosures

L. Liberale and G.G. Camici are coinventors on the International Patent WO/2020/226993 filed in April 2020; the patent relates to the use of antibodies blocking interleukin-1α to reduce various sequelae of ischemia-reperfusion injury to the central nervous system. G.G. Camici received financial support from the Alfred and Annemarie von Sick Grants for Translational and Clinical Research Cardiology and Oncology and the Swiss Heart Foundation. L. Liberale has received financial support from the Swiss Heart Foundation and the Novartis Foundation for Medical-Biological Research outside this project. S. Ministrini has received financial support from the Swiss Heart Foundation, outside this project. Y.M. Puspitasari has received financial support from the Forschungskredit Candoc grant of the University of Zurich and the Swiss Life Foundation for Public Health and Medical Research, outside this project. S. Bengs has received financial support from the Swiss Heart Foundation and the Hartmann Müller Foundation outside this project. T.F. Lüscher holds leadership positions at the European Society of Cardiology, the Swiss Heart Foundation, and the Foundation for Cardiovascular Research—Zurich Heart House and the London Heart House. T.F. Lüscher received institutional educational and research grants outside this work from Abbott, Amgen, AstraZeneca, Boehringer Ingelheim, Daiichi Sankyo, Eli Lilly, Novartis, Novo Nordisk, Sanofi, and Vifor. Since Q2, T.F. Lüscher does not accept any consulting fees from the industry. The other authors report no conflicts.

### Supplemental Material

Supplemental Methods

Table S1

Figure S1

Major Resources Table

ARRIVE Guidelines
